# Weed resistance to synthetic auxin herbicides

**DOI:** 10.1002/ps.4823

**Published:** 2018-02-15

**Authors:** Roberto Busi, Danica E Goggin, Ian M Heap, Michael J Horak, Mithila Jugulam, Robert A Masters, Richard M Napier, Dilpreet S Riar, Norbert M Satchivi, Joel Torra, Phillip Westra, Terry R Wright

**Affiliations:** ^1^ Australian Herbicide Resistance Initiative, School of Agriculture and Environment University of Western Australia Perth Australia; ^2^ International Survey of Herbicide‐Resistant Weeds Corvallis OR USA; ^3^ Monsanto Company St. Louis MO USA; ^4^ Department of Agronomy Kansas State University Manhattan KS USA; ^5^ Dow AgroSciences LLC Indianapolis IN USA; ^6^ School of Life Sciences University of Warwick Coventry UK; ^7^ Department of Horticulture, Botany and Gardening University of Lleida Lleida Spain; ^8^ Department of Bioagricultural Sciences and Pest Management Colorado State University Fort Collins CO USA

**Keywords:** synthetic auxin herbicides, herbicide resistance, resistance mechanisms, herbicide resistance mitigation, herbicide‐tolerant crops

## Abstract

Herbicides classified as synthetic auxins have been most commonly used to control broadleaf weeds in a variety of crops and in non‐cropland areas since the first synthetic auxin herbicide (SAH), 2,4‐D, was introduced to the market in the mid‐1940s. The incidence of weed species resistant to SAHs is relatively low considering their long‐term global application with 30 broadleaf, 5 grass, and 1 grass‐like weed species confirmed resistant to date. An understanding of the context and mechanisms of SAH resistance evolution can inform management practices to sustain the longevity and utility of this important class of herbicides. A symposium was convened during the 2nd Global Herbicide Resistance Challenge (May 2017; Denver, CO, USA) to provide an overview of the current state of knowledge of SAH resistance mechanisms including case studies of weed species resistant to SAHs and perspectives on mitigating resistance development in SAH‐tolerant crops. © 2017 The Authors. *Pest Management Science* published by John Wiley & Sons Ltd on behalf of Society of Chemical Industry.

## 1 INTRODUCTION

**Figure 1:**
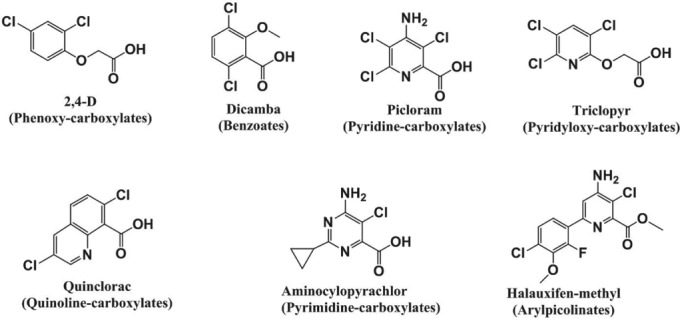
Representative herbicide structures for the seven synthetic auxin herbicide chemotypes.

Herbicides classified as synthetic auxins (HRAC group O)^1^ mimic the naturally occurring plant hormone, indole‐3‐acetic acid (IAA). The first herbicide with this mode of action, 2,4‐D, has been broadly and intensively used for more than 70 years.^2^ Synthetic auxin herbicides (SAHs) are most commonly used to selectively control broadleaf weeds in grass crops, but the SAHs, quinclorac^3^, ^4^ and florpyrauxifen‐benzyl, control some grasses and sedges. SAHs are grouped into several subclasses that include: (1) phenoxy‐carboxylates, (2) benzoates, (3) pyridine‐carboxylates, (4) pyridyloxy‐carboxylates, (5) quinolone‐carboxylates. (6) pyrimidine‐carboxylates, and (7) arylpicolinates (Table 1). Each subclass has a distinct chemical structure (Fig. 1). SAHs have been used commercially since the introduction of 2,4‐D in 1945 up to the present with the introduction of florpyrauxifen‐benzyl in 2018 (Table 1). The introduction of 2,4‐D for agricultural uses revolutionized weed management and gave rise to sustained innovation that resulted in the discovery and development of several novel SAHs.^2^, ^5^, ^6^

**Table 1:** Alignment of selected herbicides within the synthetic auxin herbicide mode of action by subclass (HRAC group O)

Subclass	Herbicide	Year of introduction*
Phenoxy‐carboxylates	2,4‐D	1945†
	2,4‐DB	1944
	MCPA	1950
	MCPB	1960
	Dicloprop	1961
Benzoates	Dicamba	1963
Pyridine‐carboxylates	Picloram	1963
	Clopyralid	1977
	Aminopyralid	2005
Pyridyloxy‐carboxylates	Triclopyr	1979
	Fluroxypyr	1985
Quinoline‐carboxylates	Quinclorac	1989
	Quinmerac	1993
Pyrimidine‐carboxylates	Aminocyclopyrachlor	2010‡
Arylpicolinates	Halauxifen‐methyl	2015
	Florpyrauxifen‐benzyl	2018§

* Pesticide Properties DataBase (PPDB), Agriculture and Environment Research Unit, University of Hertfordshire, Hatfield AL10 9AB, UK, http://sitem.herts.ac.uk/aeru/ppdb/en/atoz.htm

† Peterson *et al*.^2^

‡ https://ofmpub.epa.gov/apex/pesticides/f?p=CHEMICALSEARCH:3:::NO:1,3,31,7,12,25:P3_XCHEMICAL_ID:1192.

§ Estimated first product launch.

**Figure 2:**
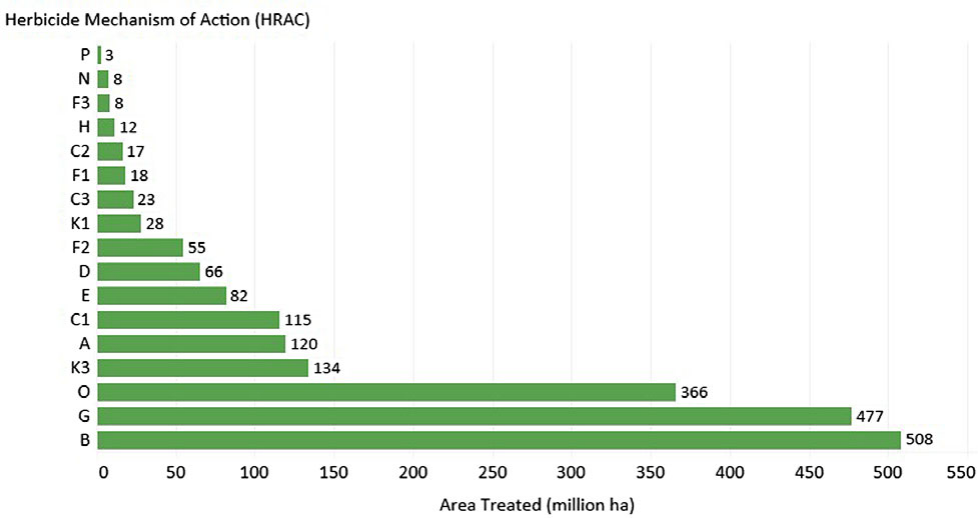
Area (×10^6^ ha) treated with the top 17 herbicide modes of action (HRAC groups) reported in 2014 (Dow AgroSciences proprietary sources, 2014).

**Figure 3:**
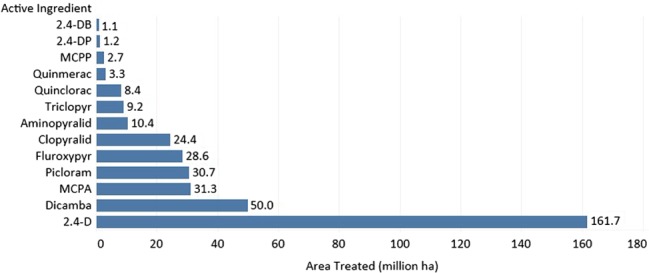
Area (×10^6^ ha) treated with specific synthetic auxin herbicide active ingredients reported in 2014 (Dow AgroSciences proprietary sources, 2014).

**Figure 4:**
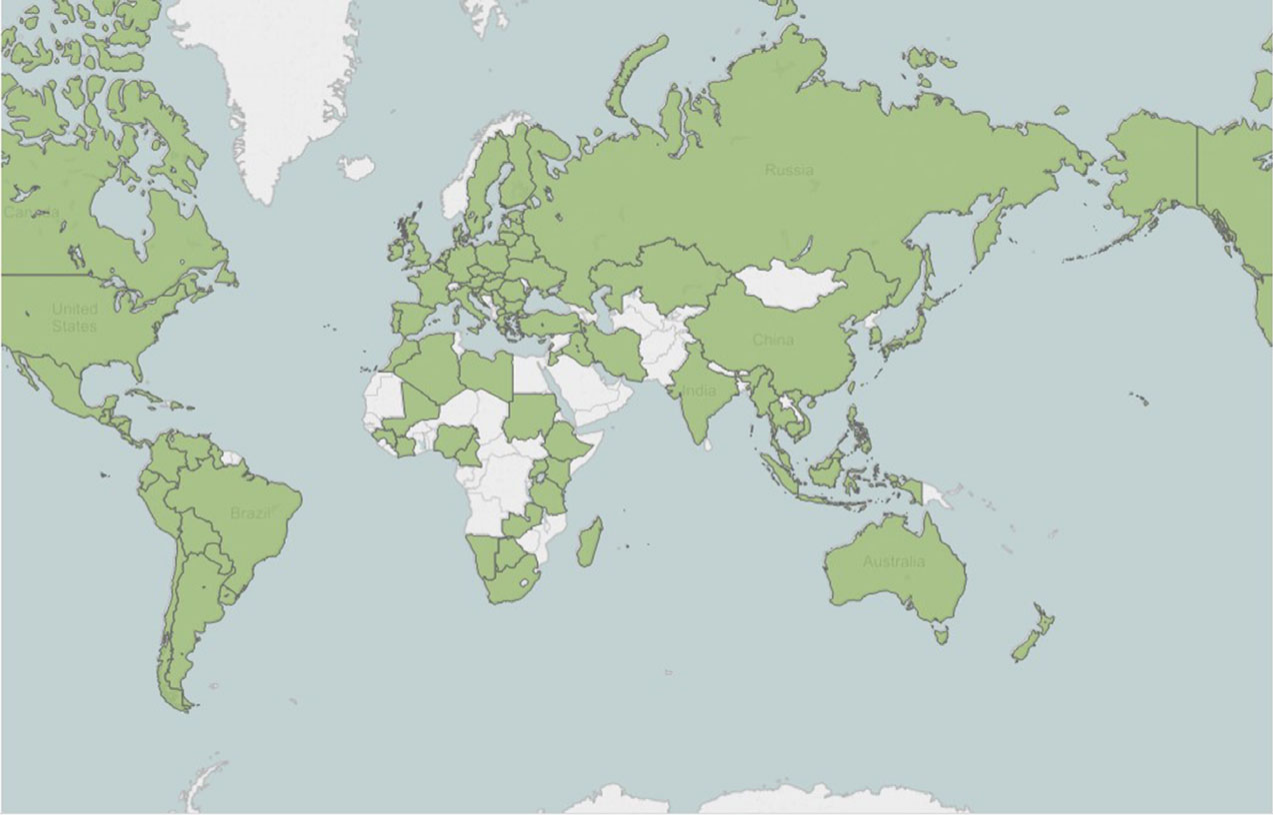
Countries (in green) where 2,4‐D use occurs (Dow AgroSciences proprietary sources, 2014). There are no reports of 2,4‐D use in countries shaded in white.

SAHs rank third (366 × 10^6^ ha) behind ALS inhibitor (508 × 10^6^ ha) and EPSP synthase‐inhibitor (477 × 10^6^ ha) herbicides (Fig. 2) in the area treated globally (Dow AgroSciences proprietary sources, 2014). The herbicide, 2,4‐D, is used on 161.7 × 10^6^ ha globally and is the most broadly used SAH, followed by dicamba (50.0 × 10^6^ ha) and 2‐methyl‐4‐chlorophenoxyacetic acid (MCPA) (31.3 × 10^6^ ha) (Fig. 3). The utility of 2,4‐D is reflected in the area treated and the large number of countries in which it is used (Fig. 4).

To sustain the utility of the SAHs, it is critical to increase our knowledge of mechanisms of resistance, and how selection and subsequent evolution of weed species resistant to SAHs has occurred. A symposium, ‘Weed Resistance to Synthetic Auxin Herbicides’ was convened during the Global Herbicide Resistance Challenge meeting in Denver, Colorado, USA in May 2017. Topics of the symposium included: evolution of weed species resistance to SAHs; mechanisms of SAH resistance evolution in well‐characterized weed species; SAH perception, transport and metabolism; recent innovation in SAH discovery; and managing resistance in SAH‐tolerant crops.

## 2 EVOLUTION OF WEED SPECIES RESISTANCE TO SYNTHETIC AUXIN HERBICIDES

**Figure 5:**
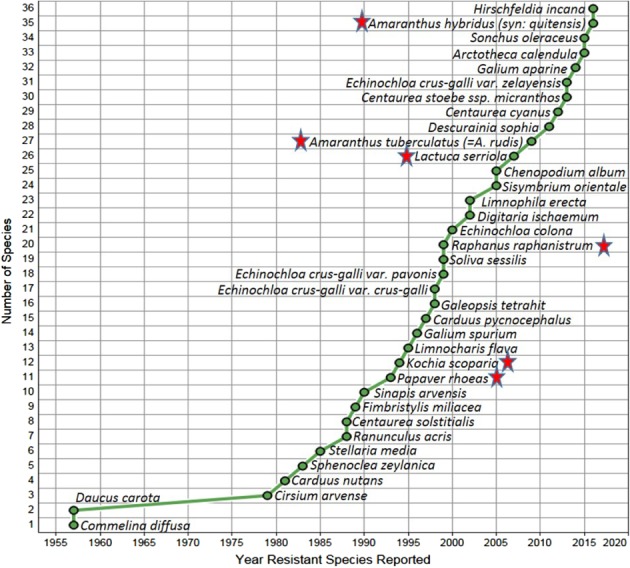
Weed species with reported resistance to synthetic auxin herbicides as of 2017.^7^ Weeds of economic importance (those that are established, spreading, and requiring a change in control tactics) exhibiting resistance to synthetic auxin herbicides are denoted by red stars.

In 1957, the first cases of 2,4‐D resistance were reported in climbing dayflower (Commelina diffusa Burm. f.) in Hawaii and wild carrot (Daucus carota L.) in Canada.^7^ According to the International Survey of Herbicide Resistant Weeds,^7^ there are now 36 SAH‐resistant weed species (30 broadleaf, 5 grass, and 1 grass‐like weed species) (Fig. 5). The five grasses that include smooth crab grass [Digitaria ischaemum (Schreb.) Schreb. ex Muhl.] and four Echinochloa species, (E. crus‐galli [(L.) P. Beauv.], E. crus‐pavonis [(Kunth) Schult.], E. zelayensis [(Kunth) Schult.], and E. colona (L.) Link), have evolved resistance to quinclorac, which has a proposed cyanide‐mediated mechanism of action on grasses that is distinct from that of SAHs.

Weeds of economic importance (those that are established, spreading, and requiring a change in control tactics) exhibiting resistance to SAHs include 2,4‐D‐ and MCPA‐resistant wild radish (*Raphanus raphanistrum* L.) in Australia, phenoxy herbicide‐resistant corn poppy (*Papaver rhoeas* L.) in Europe, dicamba‐resistant kochia [*Kochia scoparia* (L.) A.J. Scott] in Canada and the USA, prickly lettuce (*Lactuca serriola* L.) resistant to 2,4‐D, dicamba, and MCPA in the USA.^7^ Additionally, tall waterhemp [*Amaranthus tuberculatus* (Moq.) Sauer] biotypes from Nebraska and Illinois and smooth pigweed [*A. hybridus* L. (syn.: *A. quitensis* Kunth)] in Argentina were determined to be resistant to certain SAHs.^7^ Of lesser economic importance are 2,4‐D‐resistant wild carrot (*Daucus carota* L.) in Canada and the USA, 2,4‐D‐resistant musk thistle (*Carduus nutans* L.) and Italian thistle (*Carduus pycnocephalus* L.) in New Zealand, and multiple SAH‐resistant wild mustard (*Sinapis arvensis* L.) and quinclorac‐resistant false cleavers (*Galium spurium* L.) in Canada.^7^

**Figure 6:**
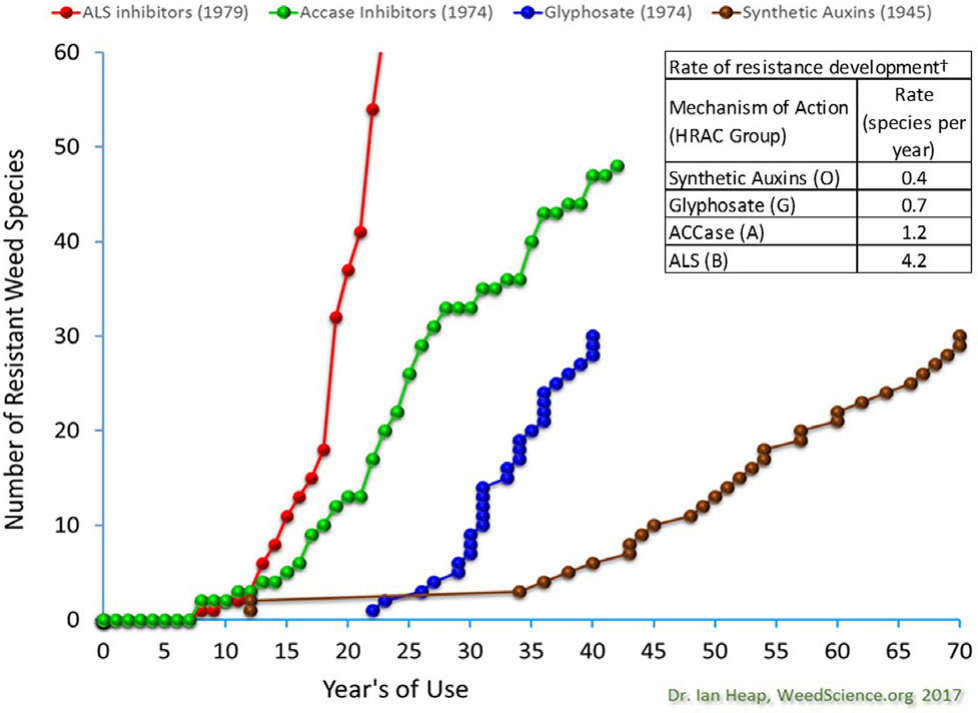
Number of weed species with reported resistance by year after introduction of ALS inhibitors (HRAC group B), ACCase inhibitors (HRAC group A), glyphosate (HRAC group G), and synthetic auxin^*^ (HRAC group O) herbicides through 2017^1^ and ranked by the relative rate of weed resistance development. Rate of weed resistance development is estimated by dividing number of weed species reported resistant by the number of years since the mode of action was introduced. For example, 159 weed species have been confirmed to be resistant to ALS inhibitor herbicides over a span of 38 years, which is about 4.2 species per year. ^*^The resistance development time line for the synthetic auxin mode of action only includes broadleaf weeds and not the 5 grass species resistant to quinclorac and 1 grass‐like species resistant to 2,4‐D listed on http://weedscience.org.

Considering the extent of selection pressure imposed by widespread use of SAHs, the incidence of weed resistance is lower when compared with other herbicide modes of action, particularly acetyl Co‐A carboxylase (ACCase)‐ and acetolactate synthase (ALS)‐inhibiting herbicides (Fig. 6). There are relatively few cases of weed resistance to SAHs that have had widespread adverse impact on agricultural production, despite these herbicides being in use longer than all other herbicide modes of action (Fig. 5).^7^

This low incidence of resistance can be attributed to several factors including: (1) potential multiple sites of action of these herbicides;^8^, ^9^ (2) a few cases of resistance conferred by recessive genes^10^, ^11^ that tend to spread more slowly than a dominant trait; and (3) reduced fitness of resistant phenotypes in the presence of herbicide and crop competition.^12^ Occurrence of cross‐resistance in weeds^13^ can be a challenge for the development of management strategies. A more thorough understanding of resistance mechanisms to SAHs should improve resistance management practices and extend their robust utility.

## 3 MECHANISMS OF SYNTHETIC AUXIN HERBICIDE RESISTANCE IN WELL‐CHARACTERIZED WEED SPECIES

### 3.1 Corn poppy (Papaver rhoeas)

Corn poppy (*Papaver rhoeas*) is the most common broadleaf weed in winter cereals in Europe.^14^ This obligate cross‐pollinated weed species is difficult to control because of high seed production, highly persistent seed banks and extended periods of germination.^14^ With the appearance and spread of herbicide resistance, corn poppy is becoming a more troublesome weed, particularly in southern Europe. Phenoxy‐carboxylate (2,4‐D and MCPA)‐resistant and ALS inhibitor (tribenuron‐methyl)‐resistant *P. rhoeas* biotypes have been reported over the last 10 years in Spain, France and Greece.^7^ In Spain, resistant populations can contain biotypes cross‐resistant to other phenoxy‐carboxylates, benzoates or pyridine‐carboxylates.^15^, ^16^ Few studies have been conducted to reveal the mechanisms and genes involved in resistance of corn poppy to SAHs.^15^, ^17^

Lack of 2,4‐D translocation in resistant plants could contribute to their resistance response.^15^ Additionally, ethylene production in susceptible plants treated with 2,4‐D was four‐ to eightfold greater than in resistant plants. It appears that 2,4‐D may not reach its nuclear protein receptor complex in resistant plants resulting in repression of auxin‐responsive genes, some of which are responsible for ethylene production.^4^, ^15^ Accumulation of ethylene can inhibit photosynthesis and produce H_2_O_2_ and reactive oxygen species that lead to plant death.^18^, ^19^

The presence of 2,4‐D metabolites was detected in shoots and roots 48 h after application in resistant *P. rhoeas* populations with impaired translocation.^17^ By 168 h after treatment, no 2,4‐D remained and only metabolites with HPLC retention times ascribed to hydroxylated 2,3‐D and 2,5‐D were detected. Treatment with malathion, a cytochrome P450 inhibitor, reversed the phenotype from resistant to susceptible. This suggests that cytochrome P450 enhances 2,4‐D degradation in these resistant populations.^17^ Reduced translocation and increased herbicide degradation may both contribute to corn poppy resistance, although which is the primary mechanism remains unknown. Polar metabolites, which are typically less phloem‐mobile than 2,4‐D, could decrease translocation.^2^ Others have found that reduced translocation occurs before herbicide degradation.^15^, ^17^ Differential metabolism, ethylene response, and translocation may be interdependent or separate mechanisms that have not been elucidated.

There are biotypes of corn poppy resistant to ALS‐inhibiting and phenoxy‐carboxylate herbicides that can degrade imazamox.^20^ It is unknown whether the same cytochrome P450 degrades herbicides with these modes of action or if other enzymes could be involved. The herbicide, 2,4‐D, was found to induce glycoside hydrolase (GH3) and glutathione S‐transferase (GST3) expression in susceptible and resistant *P. rhoeas* plants, thus increased expression of these enzymes did not appear to be involved in resistance to 2,4‐D.^21^ There is a need to identify the genes and to more fully understand the mechanisms involved in corn poppy herbicide resistance.

### 3.2 Kochia (Kochia scoparia)

Kochia is a summer annual weed that infests cropland and non‐cropland throughout the Great Plains of North America. It emerges in early spring. The protogynous flower biology ensures a high level of outcrossing that contributes to the high genetic variability found in kochia.^22^ Mature seeds do not have a hard outer coat and exhibit little dormancy.^23^ Kochia has evolved resistance to atrazine, dicamba, glyphosate and several ALS inhibitor herbicides.^24^, ^25^, ^26^, ^27^, ^28^, ^29^

Dicamba‐resistant kochia was first observed in the 1990s in Colorado and Nebraska.^30^, ^31^ By 2016, herbicide‐resistant biotypes had become common, especially in wheat–fallow fields in Colorado and Kansas. To date, no cross‐resistance to fluroxypyr has been observed in Colorado, although there is a report from Montana.^27^ Using advanced selection techniques over a 25‐year period, a kochia accession 9425 was developed that exhibited a 30‐fold increase in dicamba resistance compared with a sensitive accession 7710. The trait was characterized as dominant or semi‐dominant.^32^ The 9425 accession showed reduced translocation of dicamba compared to susceptible accessions. RNA‐seq revealed a unique set of genes impacting auxin transport that were upregulated in accession 9425, which may explain reduced dicamba translocation in resistant plants.^33^

A small number of putative fluroxypyr‐resistant kochia populations have been found.^27^ Although fluroxypyr has been an effective and reliable herbicide for kochia control in a variety of settings, it is not viewed as a ‘standalone’ product due to its narrow spectrum of weed control. As resistance to other herbicides becomes more widespread in kochia, it will be important to steward the growing use of fluroxypyr for control of kochia to avoid resistance evolution.

### 3.3 Prickly lettuce (Lactuca serriola)

Prickly lettuce is an annual, winter annual or biennial weed in the Asteraceae family that is problematic in the Pacific Northwest, USA. This plant has a deep tap root that helps it survive drought conditions. Besides competing for moisture, space and nutrients with crops, sticky white latex produced by plants can clog harvesting equipment and increase grain moisture content. Flower buds mixed with grain during harvest operations are hard to screen and reduce grain quality.

Almost 80% of wheat produced in the Pacific Northwest receives at least one application of a SAH. Prickly lettuce plants that survived two applications of glyphosate at 840 g a.e./ha plus 2,4‐D at 540 g a.e./ha were identified near Pullman, WA in summer 2004.^34^ Further investigation showed differential 2,4‐D response was heritable and lack of control was not a result of glyphosate antagonism. Resistant plants appeared to be injured by 2,4‐D 2 to 3 weeks after treatment with regrowth initiating from the crown 3 weeks after treatment. The level of resistance was eight‐ to nine‐fold greater than that of a susceptible biotype based on 50% growth reduction assessments. This 2,4‐D‐resistant biotype appears to have evolved cross‐resistance to other SAHs in the phenoxy‐carboxylate and benzoate subgroups, including MCPA and dicamba.^35^

Inheritance of 2,4‐D resistance in prickly lettuce was governed by a single codominant gene.^36^ Biokinetic studies using ring‐labeled 14C‐2,4‐D were conducted to understand this mechanism of 2,4‐D resistance. At 96 h after application, the resistant biotype absorbed less 2,4‐D and retained more radioactivity in the treated leaf compared with the susceptible biotype. No difference in rate of metabolism of 2,4‐D was observed in the treated leaf or crown between resistant and susceptible biotypes.

Growth was stimulated in the susceptible biotype during the first 4 days after treatment, but not in the resistant biotype.^36^ Beyond 4 days, growth of the susceptible biotype almost ceased, while the resistant biotype continued to grow. Continued growth of the resistant biotype could be the result of reduced deregulation by an altered auxin signaling response. Perhaps altered auxin signaling and maintenance of normal growth in resistant biotypes could have reduced 2,4‐D uptake and translocation in the resistant prickly lettuce biotype. The precise gene responsible has not been determined.^36^ Transcriptomics and targeted gene expression investigation could help identify potential gene(s) responsible for altering auxin signaling response in 2,4‐D‐resistant prickly lettuce biotypes.

### 3.4 Wild radish (Raphanus raphanistrum)

Wild radish is the most problematic dicot weed in southern Australian cropping systems, costing growers at least $57 million (AUD) per year in lost crop yield and increased weed control expenses.^37^, ^38^ In Western Australia, this species has developed widespread resistance to the sulfonylurea and imidazolinone classes of ALS‐inhibiting herbicides,^39^ which has prompted growers to rely on SAHs, particularly 2,4‐D. In 1999, the first two cases of field‐evolved 2,4‐D resistance in wild radish were identified.^40^ Subsequent random weed population surveys carried out in 2003, 2010 and 2015 revealed that the proportion of populations containing 2,4‐D‐resistant plants increased from 60% of surveyed populations in 2003 to 74% in 2010.^39^, ^41^ Levels of resistance remained unchanged in 2015.

Phenotypic characterization of two wild radish populations collected from the field in 2001 and 2002 showed about a 20‐fold increase in resistance compared with a susceptible population. The traits conferring 2,4‐D resistance in these two populations segregated at one major locus, and were nuclear‐inherited and incompletely dominant.^42^ The 2002 population and a population collected in 2010 exhibited highly restricted translocation of ^14^C‐labeled 2,4‐D out of the treated leaf.^43^ The restriction of 2,4‐D translocation, which could be mimicked in susceptible plants by application of the auxin efflux inhibitor 1‐N‐naphthylphthalamic acid, may be due to loss of function of an ATP‐binding cassette type B (ABCB)‐type long‐distance auxin efflux transporter in the resistant plants. The primary resistance mechanism in wild radish appears to be reduced 2,4‐D translocation.

The 2,4‐D‐resistant biotypes collected from populations in 1999, 2001 and 2002 were also resistant to MCPA.^44^ A study that included the MCPA‐resistant biotypes in the 1999 population revealed an inheritance pattern similar to that observed in 2,4‐D‐resistant biotypes.^44^ In experiments measuring seedling root elongation, the 2002 population was resistant to 2,4‐D, MCPA, mecoprop, dicamba and 1‐naphthylacetic acid, whereas the 2010 population was only resistant to the phenoxy acetic acids, 2,4‐D and MCPA.^44^ This suggests a difference in auxin perception and/or signal transduction among resistant biotypes. A genome‐wide transcriptomics study of the 2002 population revealed a rapid upregulation of auxin‐induced transcriptional repressors and defense genes that was not observed in the susceptible population (Goggin DE *et al*., unpublished). This is likely a second resistance mechanism, since some populations that were highly resistant to 2,4‐D translocated ^14^C‐labeled 2,4‐D out of the treated leaf as efficiently as susceptible plants (Goggin DE *et al*., unpublished).

Biomass of plants grown in competition with wheat and treated with the recommended rate of 2,4‐D was reduced by 75% compared with 2,4‐D‐treated plants grown without competition.^12^ It would be useful to determine plant fitness in the absence of herbicide treatment in those populations with varying levels of cross‐resistance and herbicide translocation, and/or different levels of expression of resistance genes. This information could identify appropriate non‐chemical practices, such as early crop planting and use of highly competitive crop varieties, which could contribute to more effective resistance management strategies.

Finding alternative molecules that interfere with the genes or gene products involved in altering SAH response of resistant plants or those capable of re‐activating normal translocation of the herbicide could prolong the utility of the SAHs in wild radish‐infested crops. To date, there is no evidence of metabolic detoxification of 2,4‐D (either cytochrome P450‐dependent or ‐independent) in resistant wild radish populations.^43^ Continued vigilance is required to detect evolution of populations with metabolic resistance so resistance management practices can be adjusted to better control these populations.

## 4 SYNTHETIC AUXIN HERBICIDE PERCEPTION, TRANSPORT AND METABOLISM

### 4.1 Potential target sites for resistance to auxin herbicides: auxin receptors

In the case of SAHs, the candidate mechanisms of resistance can include target site modifications (auxin receptors or auxin‐specific transporters) and non‐target site mechanisms such as other transporters and enzymes that metabolize SAHs. One major source of resistance to herbicides is target site resistance. Target site resistance frequently arises from mutations in the active sites of the proteins to which the herbicide normally binds, rendering the protein insensitive to certain herbicides as non‐native compounds. No incidence of resistance arising from SAHs applied in the field has been traced back to any of the core signaling target sites. However, mutations in many of these proteins can confer resistance to SAHs, as demonstrated by the fact that most auxin signaling and transport proteins were identified from screens for Arabidopsis mutants tolerant to applied SAHs.^45^, ^46^

Scientists have identified a number of putative auxin receptor proteins,^47^ but the receptor TIR1 is the archetype.^48^, ^49^ In Arabidopsis, TIR1 is part of a family of six receptors, with the other five known as Auxin F‐Box proteins (AFB1–5). Based on genomic duplication, the family is known to comprise three pairs: TIR1 and AFB1, AFB2 and AFB3, and AFB4 and AFB5. There is a large degree of sequence similarity between all the receptors and a high level of functional redundancy. A loss of function mutation in any one of these genes results in little change of plant phenotype compared with a wild‐type and only with stacked mutations will plant growth and development be affected. 50

**Figure 7:**
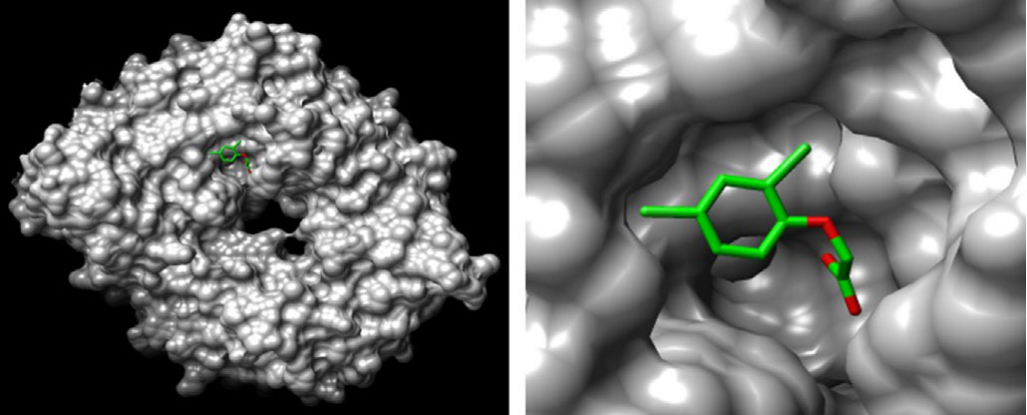
Structure of auxin receptor TIR1 with bound 2,4‐D. The receptor protein surface (grey) viewed from above (left), with 2,4‐D (colored for heteroatoms) shown docked at the base of the deep auxin binding pocket. A close‐up looking down the binding pocket (right) shows how well the shape of 2,4‐D conforms to the space at the base of the site. The image was produced using Chimera from protein database file 2P1N.

The TIR1 receptor structure was determined by crystallization.^51^ The TIR1 mechanism of action was resolved by studying the characteristics of TIR1 binding auxins with the co‐receptor proteins AUX/IAA (Fig. 7).^52^ Pharmacophoric maps have been developed by screening a wide range of auxins and auxin‐like molecules, and these maps define the detailed characteristics of active ligands for each receptor.^52^

The TIR1 and AFB1–5 auxin receptors vary little in their sequences and there is a high degree of sequence conservation across the plant kingdom, especially in amino acids that line the binding site.^53^ The implication is that a mutation would compromise plant fitness and any deviation from the wild‐type would be disadvantageous. If this is correct, then target site resistance is unlikely to arise. The redundancy of the receptor family does build resiliency into the plant system such that a loss of efficacy in one receptor could be compensated for by the others. The loss of fitness holds only for sensitivity to the endogenous auxin, IAA. Thus, it is important to understand if there is incipient resistance within the TIR1 family, and whether different members of the receptor family are differentially selective for different SAHs. There is clear evidence that AFB5 differs from TIR1 in selectivity for different SAHs, with AFB5 receptors being the primary site of action for the picolinate herbicides,^9^, ^52^ and there is also evidence that an Arabidopsis AFB5 mutant line insensitive to picloram does not have altered sensitivity to 2,4‐D or IAA.^8^, ^54^ Therefore, it is possible that a mutation could arise in AFB5 homologues in weed populations that could confer resistance to picolinate herbicides, without compromising sensitivity to endogenous auxin or plant fitness. Stewardship of picolinate herbicides needs to account for and prevent such an outcome.

Co‐receptor AUX/IAA proteins should be considered because they bind on top of auxin once it is inside the recognition pocket to complete the co‐receptor complex (Fig. 7).^51^ Different AUX/IAAs bind with different auxin concentrations^52^ and it is possible that mutations in co‐receptors will contribute to SAH resistances. Target site resistance based on auxin receptors and co‐receptors is possible even though such resistance has not yet been identified. Should field resistance arise to one class of SAHs, it is likely that SAHs based on different scaffolds will remain active because of redundancy in the receptor family.

### 4.2 Potential target sites for resistance to auxin herbicides: auxin transporter proteins

**Figure 8:**
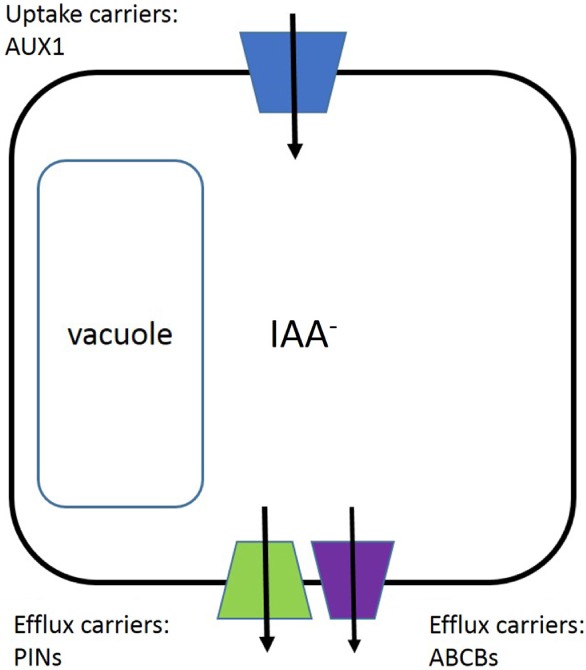
Arrangement of auxin transport proteins. The plasma membrane of plant cells contains uptake carriers such as AUX1, which facilitate accumulation of IAA and 2,4‐D inside the cytoplasm. To drive polar auxin transport, two distinct families of auxin efflux proteins are situated on the polar basal sides of the cell and in the plasma membrane.

Transport proteins can confer both target‐site and non‐target‐site resistance (Fig. 8).^55^ For auxins, selective uptake into plant cells is conferred primarily by the AUXin resistant 1 (AUX1) carrier, although the LAX proteins (Like AuX1) contribute in certain plant tissues.^56^ The initial AUX1 mutation was isolated on the basis of its resistance to 2,4‐D^57^ so it is known that this target site is vulnerable. The structure for AUX1 is not yet described, but there is a radiolabeled 2,4‐D accumulation assay with tobacco cell cultures that can measure AUX1 activity.^58^ A diversity of auxin‐like structures have been screened and a pharmacophoric map of AUX1 selectivity is expected.^59^ Several SAH scaffolds are not substrates for AUX1, and yet the compounds are active as SAHs. Consequently, it seems unlikely that SAH resistance will arise from AUX1 mutations or from reductions to AUX1 abundance in the plasma membrane.

Selective auxin efflux gives rise to polar auxin transport and contributes to the establishment and maintenance of plant cell polarity.^60^ There are two important efflux carrier protein families, the PIN‐FORMED (PIN) and the ABCB proteins. The PINs are plant‐specific and ABCBs are part of a family of ATP‐driven transporters that are responsible for drug resistance in medicine.^61^ Given that both PINs and ABCBs are complex membrane‐bound proteins, the structures are currently unknown. In addition, there are no widely used transport assays suited to investigate their pharmacology. A few selective inhibitors of transport are known,^62^ and one, naphthylphthalamic acid, is registered as an herbicide.

It is more challenging to evaluate the potential of efflux proteins as sites for resistance on the basis of available biochemical knowledge. Upregulation of either efflux carrier would likely accelerate extrusion of substrate auxins from cells, which may confer resistance by removing herbicide. The PINs are dominant when considering endogenous auxin concentrations and gradients.^60^ Little is known about PIN selectivity to different SAHs, but PIN activity is known to be self‐reinforcing, so increasing auxin doses will increase activity, probably through recruitment or reduced loss of PINs to the plasma membrane.^63^ High doses of SAH could promote PIN recruitment, but how they affect extrusion and resistance will depend on the selectivity of the PIN protein for that herbicide. More knowledge of the mechanism of auxin pumping and of the pharmacological selectivity of the PIN proteins would help reveal the PIN role in SAH action.

The ABCB transport proteins are well characterized in non‐plant eukaryotic systems and there is a growing understanding about the importance of ABCB transport proteins in plants.^55^ A preliminary homology model for plant ABCBs has been presented, although insufficiently refined to help our understanding of substrate selection.^64^ There are plant ABCBs linked specifically to auxin efflux and to physiological responses dependent on their correct function.^64^ The family has a wide range of small molecule substrates, and alterations in ABCB abundance at the plasma membrane leads to resistance to small molecule drugs.^61^ A better understanding of this family of plant transport proteins is relevant to avoiding SAH resistance evolution. Resistance to 2,4‐D has been circumstantially associated with ABCB transporter activity, although impaired long‐distance transport and not increased cellular efflux, which consequently reduced accumulation inside cells, appeared to be the basis of resistance.^43^

### 4.3 Potential non‐target site resistance mechanisms to auxin herbicides: auxin metabolism

Non‐target site resistance to agrochemicals has frequently been associated with their metabolism by enzymes into harmless products.^65^ The major route for deactivating the endogenous auxin, IAA, is by conjugation.^66^ The enzymes responsible for conjugating IAA may be considered candidates for deactivating SAHs and a potential source for resistance. There are three principal routes for IAA conjugation in plants, including conjugation of amino acids to the auxin by GH3 proteins,^67^ conjugation of sugars by glucosyltransferases,^68^ and conjugation of glutathione by GST3.^69^ Structures have been defined for GH3 proteins bound to IAA and salicylic acid.^67^ In principle, conjugating enzymes could be sources of resistance, but it is unlikely that any one enzyme would recognize all the various SAH scaffolds as substrates.

Oxidation of small molecules, often by cytochrome P450‐type enzymes, may be a mechanism for detoxifying herbicides^65^ and other agrochemicals, including insecticides.^70^ Plant resistance to quinclorac has been associated with these enzymes.^71^ The primary catabolic pathway for the endogenous auxin IAA was identified as an IAA oxidase,^72^ not a cytochrome P450. It is not known if this oxidase will also use SAHs as substrates. Although endogenous IAA oxidase is a possible source of resistance, resistance to the broad diversity of SAHs seems unlikely. Oxidation has become the latest mechanism used in development of dicamba‐ and 2,4‐D‐tolerant crops.^73^, ^74^ In these cases, two distinct oxidases of bacterial origin were introduced to confer resistance.

## 5 RECENT INNOVATIONS IN SYNTHETIC AUXIN HERBICIDE DISCOVERY

The discovery of aminopyralid in the late 1990s prompted an intensive structure–activity relationship (SAR) assessment of the pyridine‐carboxylate SAHs at Dow AgroSciences. This led to the discovery of a novel chemical family of SAHs, the arylpicolinates, which are derivatives of pyridine carboxylic acids. The acidic functionality of SAHs is critical for their mobility in the phloem and for accumulation inside cells through ion trapping. For the pyridine‐carboxylate herbicides clopyralid, picloram, aminopyralid, and arylpicolinate herbicides [halauxifen‐methyl (Arylex™ Active) and florpyrauxifen‐benzyl (Rinskor™ Active)], the carboxylic acid functionality is involved in key binding interactions at the TIR1 and AFB1–5 receptors.^75^ The arylpicolinates are formulated as esters and it is likely that these esters are rapidly hydrolyzed to free carboxylic acids in the plant.

**Figure 9:**
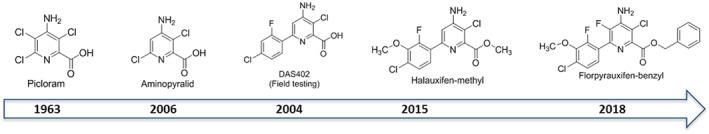
Herbicidal activities of picloram and aminopyralid were discovered 43 years apart. A thorough structure–activity relations exploration after aminopyralid discovery led to the novel arylpicolinate synthetic auxin herbicide class. The long soil half‐life of the highly active arylpicolinate analog (DAS402) was reduced by introducing a methoxy group to the 3‐position of the phenyl tail resulting in the analog, halauxifen‐methyl (Arylex™ Active). Further exploration resulted in the discovery of florpyrauxifen‐benzyl (Rinskor™ Active).

The arylpicolinates have unique herbicidal activities that include increased efficacy at relatively low use rates (30 g acid equivalent (a.e.)/ha or less) compared with pyridine carboxylic acid herbicides. The analog DAS402 (Fig. 9), exhibited an average herbicidal efficacy significantly greater than aminopyralid, but the soil half‐life of DAS402 was deemed too long (> 240 days) to meet the regulatory requirements for anticipated commercial uses. Efforts to increase degradation rate in soil included the addition of a metabolic handle into the structure. This led to the discovery of methyl 4‐amino‐3‐chloro‐6‐(4‐chloro‐2‐fluorophenyl‐3‐methoxy) picolinate (halauxifen‐methyl) (Fig. 9). Halauxifen‐methyl was determined to be a potent broad‐spectrum SAH that could be safened to achieve excellent selectivity in wheat and barley.^76^, ^77^, ^78^ Halauxifen‐methyl exhibited a soil half‐life of 10 to 30 days, much shorter than DAS402. The first commercial launch of halauxifen‐methyl was in 2015 for use in cereals.

During the exploration of the halauxifen‐methyl SAR, several important features of the picolinate scaffold were identified. The 2‐carboxylic acid and the 4‐amino functional groups of the molecule were found to be essential for the expression of potency and broad‐spectrum herbicide activity and the 3‐chlorine functional group contributed further to herbicide potency. Exploration of the SAR of the 5‐position of the picolinate head of halauxifen‐methyl led to the discovery of a second arylpicolinate herbicide with commercial utility. This 5‐fluoro analog of halauxifen‐methyl exhibited potent and broad‐spectrum herbicide activity with excellent selectivity for use in rice. Its corresponding carboxylic acid (4‐amino‐3‐chloro‐6‐(4‐chloro‐2‐fluorophenyl‐3‐methoxy)‐5‐fluoropicolinic acid) had a soil half‐life in the range of 10 to 30 days. Further optimization of the ester eventually led to the development of florpyrauxifen‐benzyl^78^ for use in rice and other crops with the unique feature of providing control of the annual grass, *Echinochloa crus‐galli*, and some sedges (*Cyperus* spp). The first registration for florpyrauxifen‐benzyl was approved in 2017.

The rise of the arylpicolinate herbicides provides compelling evidence that there are rich opportunities for developing new SAHs. The high diversity of activity of the arylpicolinates adds to the long lineage of SAHs that have brought broad utility to weed management market segments including cereals, rice, rangeland, pastures, and rights‐of‐way.

## 6 MANAGING RESISTANCE IN SYNTHETIC AUXIN HERBICIDE‐TOLERANT CROPS

New herbicide formulations have been developed for use in recently developed crops tolerant to 2,4‐D, developed by Dow AgroSciences, and dicamba, developed by Monsanto. Enlist Duo™ (2,4‐D‐choline + glyphosate) and Enlist One™ (2,4‐D‐choline) herbicides were developed for the Enlist™ Weed Control System and Xtendimax (dicamba) with VaporGrip® was developed for the Roundup Ready® Xtend Crop System. Dow AgroSciences and Monsanto have developed stewardship programs designed to ensure the longevity and sustainability of these herbicide‐tolerant crop systems and to provide guidance on their integration into herbicide resistance management programs. In addition, the US Environmental Protection Agency (USEPA) is developing a regulatory framework to emphasize the need for grower adoption of herbicide resistance best management practices to further the sustainability of these crop systems.^79^

With the introduction of SAH‐tolerant crops, there has been extensive consideration of herbicide resistance management from regulatory and stewardship perspectives. These tools will aid in weed management programs and the control of some species of herbicide‐resistant weeds. Best management practices^80^ that are widely accepted to delay the selection for herbicide resistance in weed populations and to manage populations of existing resistant weeds form a basis for stewardship of these technologies. These best management practices are the focus of conditions of registration in the USA as prescribed by the USEPA.

From the 1950s to 2001, herbicide registrants had to fulfill only a few requirements in the USA regarding herbicide resistance management. After 2001, registrants were required to report confirmed herbicide‐resistant weed species to USEPA using the Adverse Effects Reporting [6(a)2] process.^81^ In addition, the USEPA indicated that registrants may voluntarily place mode of action on herbicide product labels and include a section that defines herbicide resistance and the best management practices to prevent or delay evolution of weed resistance to herbicides.^82^ In 2014, the USEPA issued for the first time conditions of registration for Enlist Duo herbicide,^83^ which included the requirements for an herbicide resistance management plan. In 2016, similar conditions of approval were issued for Xtendimax herbicide with Vaporgrip.^84^ The USEPA herbicide resistance management plans for these SAH weed management systems consist of field detection and remediation, education and information, evaluation, best management practices and reporting.

Strong collaboration among stakeholders to develop and deploy effective herbicide stewardship programs are needed to limit the onset of field‐evolved resistance and to ensure herbicide longevity and sustainability. Along with herbicide developers and registrants, farmers, regulators, extension specialists, crop advisors, dealers and retailers have roles to play in technology stewardship. The industry encourages weed management stakeholders to continue to promote the use of herbicides as part of a comprehensive weed management approach so that these valuable tools can be preserved by delaying onset of field‐evolved herbicide resistance.

## 7 CONCLUDING COMMENTS

Our purpose is to synthesize the state of the knowledge about plant resistance to SAH and to reinforce the compelling opportunities for future innovation within the SAH mode of action. The SAHs are unique and complicated in their pharmacology. Their complex mechanism of action, beneficial impacts on agricultural production, and durability are a testament to their utility in weed management systems.

The SAHs continue to be viable after more than 70 years of commercial use, partly because they have not commonly been used as the sole instrument for weed control. Selection for resistant biotypes does occur when the diversity of the tools employed is limited. Recent insights into the mechanism and site of action of SAHs, including redundant auxin receptors and complex molecular interactions between auxin‐responsive genes and cascading disruption of homoeostasis on multiple biochemical pathways, help explain why simple hypotheses for many resistant field biotypes have yielded unsatisfactory answers to questions about mechanisms of resistance. This knowledge has given credence to the proposed reasons why target site resistance has been slow to develop and may be associated with significant fitness cost .^9^ Recent investigations of new resistant weed biotypes using the latest molecular and biochemical tools provide insights into future resistance risks (in particular for non‐target site resistance mechanisms) and potential strategies for mitigating these risks as new SAHs are designed, introduced into the market and used.

A simple altered site of action with reduced affinity for SAHs has been difficult to demonstrate. Hypotheses of increased metabolism or decreased uptake into the plant often do not adequately explain biotype responses to SAHs. With insights into the interactions of chemistry, proteins, and gene regulation, and with improved analytical tools, we now understand that reduced transport can potentially explain resistance to SAHs. However, enhanced metabolism may (e.g., corn poppy) or may not (e.g., prickly lettuce and wild radish) be associated with SAH resistance. Understanding the impacts of each of these mechanisms has proven difficult because of the multiple and redundant variables in the complex auxin responsive pathways.

In our collective view, the risk of additional herbicide‐resistant biotypes developing with altered target sites (binding proteins, TIR1/AFB1–5) remains relatively low. We also believe the complex interaction with the auxin perception and responsive pathway makes resistance through overexpression of the target proteins (as seen with glyphosate‐resistant kochia and Palmer amaranth) a low probability. As SAH use continues to grow, the risk for resistance selection will also increase. Our concern is that additional sequestration and/or altered transport mechanisms of resistance (e.g. ABCB transporters, *inter alia*) may develop and spread. A significant concern could be the selection of traits causing an altered transport for SAHs potentially resulting in co‐evolution of resistance to other herbicide modes of action, as seen with multiple drug‐resistant bacteria.^85^ Differential metabolism of SAHs has been observed in some weed biotypes^17^ and understanding these resistance mechanisms is critical to developing and implementing more effective resistance management strategies.

These insights have been made possible by improved knowledge and analytical capabilities. Yet, the pharmacology of cellular and long‐distance transport mechanisms in plants are poorly understood. Pursuing greater understanding of these mechanisms is a clear opportunity for weed science and allied disciplines. The fact that after more than 70 years of use, new SAHs demonstrating unique weed control spectra and different chemical attributes continue to be discovered supports the proposition that the future is rich with opportunities for further innovation with SAHs.

Another lesson is that the entire community of weed control experts including industry scientists, academia, regulatory agencies, and farmers need to be aware and fully supportive of the implementation of best herbicide resistance management practices.^80^ ‘Diversity’ remains the mantra to sustain effective long‐term weed management. For example, the combination of soil residual herbicides of differing modes of action together with post‐emergence herbicide programs as well as non‐chemical weed management methods (i.e., cover crops, strategic tillage, crop rotations and harvest weed seed control measures) will likely keep our weed management tools viable, enable modern agriculture to thrive and provide food security for future generations.
